# Comprehensive Assessment of Rapeseed Meal as a Fish Meal Substitute in Hybrid Sturgeon (*Acipenser schrenckii* ♀ × *Acipenser baerii* ♂) Diets: Impacts on Growth Performance, Body Composition, Immunological Responses, Intestinal Histology, and Inflammatory Response

**DOI:** 10.1155/anu/6415465

**Published:** 2025-01-22

**Authors:** Wenpeng Zhang, Siyuan Liu, Shidi Wang, Huamin Wang, Kaibo Ge, Yuhong Yang, Shaoxia Lu, Shicheng Han, Haibo Jiang, Chang'an Wang, Hongbai Liu

**Affiliations:** ^1^Key Laboratory of Aquatic Animal Diseases and Immune Technology of Heilongjiang Province, Heilongjiang River Fisheries Research Institute, Chinese Academy of Fishery Sciences, Harbin, China; ^2^College of Animal Science and Technology, Northeast Agricultural University, Harbin, China; ^3^College of Fisheries and Life Science, Dalian Ocean University, Dalian, China; ^4^College of Fisheries and Life Science, Shanghai Ocean University, Shanghai, China; ^5^College of Animal Science, Guizhou University, Guiyang, China

**Keywords:** growth performance, hybrid sturgeon, immunological response, intestinal histology, rapeseed meal

## Abstract

This study aimed to assess the impact of incorporating rapeseed meal (RM) as a partial substitute for fish meal (FM) in the diet of cultured hybrid sturgeon (*Acipenser schrenckii* ♀ × *Acipenser baerii* ♂). A total of 450 juvenile hybrid sturgeon with similar weights were randomly assigned to five dietary groups, each with triplicates of 30 fish per tank. For 12 weeks, FM was replaced with varying percentages of RM (0%, 25%, 50%, 75%, and 100%). Results indicated a decreasing trend in final body weight (FBW), weight gain, and survival rates (SRs) as the ratio of RM increased. Growth performance was less affected when the substitution ratio of FM was below 50%. The replacement of FM with RM showed a decreasing trend in crude protein and ash content of sturgeon body composition and no significant effect on moisture and crude lipid content (*p* > 0.05). Essential amino acids (EAAs) in whole fish, such as methionine (Met), threonine (Thr), and lysine (Lys), increased with higher substitution rates (*p* > 0.05). The lysozyme (LZM) activities in the pyloric cecum, duodenum, and valve intestine of the sturgeon showed a decreasing trend (*p* > 0.05). Nevertheless, at a 50% substitution level, sturgeon liver superoxide dismutase (SOD) and malondialdehyde (MDA) activities reached their peak. At 100% substitution, serum alanine aminotransferase (ALT) and aspartate aminotransferase (AST) activities were significantly higher than in other groups (*p* < 0.05). At 50% substitution, sturgeon valve intestinal protease activity reached its maximum, and the duodenal villus height (VH) was not significantly different from that of the control group (morphological and structural indices were lower in the treatment groups). Gene expression of pro-inflammatory factors IL-1*β*, IL-6, IL-8, and TNF-*α* increased with the substitution ratio, while anti-inflammatory factor IL-10 showed the opposite trend. NF-*κ*B and myeloid differentiation factor 88 (MyD88) expression increased with substitution ratio, and Toll-like receptor 1 (TLR1) and Toll-like receptor 2 (TLR2) showed the opposite trend in the intestine. The results of this study suggest that replacing less than 50% of fishmeal with RM in hybrid sturgeon diets can reduce the amount of fishmeal used without compromising fish health.

## 1. Introduction

Aquaculture has experienced significant growth, producing fish products with a notable nutritional value comparable to milk, eggs, and beef, featuring high-quality proteins, vitamins, minerals, and easily digestible polyunsaturated omega-3 fatty acids essential for human consumption [1, 2]. Aquaculture heavily relies on fish meal (FM), especially for carnivorous fish, as it constitutes their primary protein source in nutrition [3, 4]. FM holds significance in aquafeeds owing to its high protein content, abundance of unsaturated fatty acids, absence of antinutritional elements, and ease of digestion and absorption [5]. In addition, it serves as a rich reservoir of indispensable amino acids, minerals, vitamins, and other vital nutrients [6]. With the rapid expansion of fish farming, there has been a notable surge in FM consumption, a trend expected to persist in tandem with ongoing growth [2]. Consequently, the quest for replacing protein sources to curtail FM usage has emerged as a paramount focus within aquaculture nutrition and feed practices. In efforts to lower feed costs, studies in recent years have explored the potential of alternative proteins, which can either partially or entirely substitute FM, with a focus on utilizing economical animal byproducts [7]. Various components like poultry byproduct meal (PBM) [8, 9], blood meal (BM) [10], meat and bone meal (MBM) [11], and hydrolyzed feather meal (HFM) [12] have garnered interest as potential alternative protein sources for fish diets. Plant-based protein sources may offer considerable advantages over animal-based proteins. Numerous research studies have showcased the effects of diverse plant protein substitutes for FM across various fish species, including fermented cottonseed meal (FCSM) [13], soybean meal (SM) [14], fermented mulberry leaf meal (FMLM) [15], pumpkin seed cake (PSC) [16], and rapeseed meal (RM) [17, 18]. RM is the residue of rapeseed oil extraction and serves as a high-quality protein source for feed production [19]. Its abundance and rich protein content render it a crucial ingredient in fish nutrition. Studies have reported that adding RM in moderate amounts to a variety of fish feeds does not significantly reduce the growth performance. For instance, substituting 22.08% and 56.25% FM with RM exhibited no adverse effects on the growth performance of juvenile Nile tilapia (*Oreochromis niloticus*) and Asian red-tailed catfish (*Hemibagrus wyckioides*) [20].

Nevertheless, RM contains various antinutritional factors (ANFs) like phytic acid, tannins, thioglucosides, and their derivatives, which detrimentally impact fish feeding rate, body composition, and the development of internal organs [17, 18, 21, 22]. Substituting elevated levels of RM has been shown to reduce the growth and feed intake in tilapia [23], catfish (*Silurus glanis*) [24], and grass carp (*Ctenopharyngodon idella*) [25]. It also negatively impacts the nonspecific immune system and antioxidant capacity of the greater yellow croaker (*Larimichthys crocea*) [26] and *Pseudobagrus ussuriensis* [27]. Additionally, adverse effects on the intestinal histomorphology and immunity of juvenile grass carp (*C. idella*) have been reported when RM replaced FM in the diet at a 54% rate [28].

The sturgeon, noted as one of the earliest surviving fishes [29], holds the status of a “living fossil” [30, 31], rendering it a significant subject for the study of biological evolution. Recognized for its superior quality, both in its flesh and caviar, the sturgeon stands as a valuable resource in fisheries [32]. To ensure sustained progress in sturgeon farming, researchers are actively enhancing and refining its feeds [29]. The main sturgeon feeds include maize bran, wheat bran, rice bran, SM, cottonseed cake, FM, and bone meal [33]. Among these, FM holds the advantage of high protein content, ease of digestion, and widespread use as a key constituent in fish feeds [34, 35]. Nonetheless, the rapid expansion of sturgeon farming has led to a scarcity of FM raw materials, causing a notable surge in prices [36, 37]. The overproduction of FM is not conducive to environmental protection [38]. Consequently, exploring alternatives to FM has emerged as a critical concern within aquatic animal nutrition and feed research [39]. Researchers have undertaken numerous experiments to determine the ideal ratio of substitute proteins in sturgeon diets. Research findings indicate that a blend of mixed plant proteins (SM, RM, cottonseed meal) substituting 30% of FM protein in hybrid sturgeon diets did not negatively impact growth performance, feed utilization, or body composition [40]. Similarly, in Siberian sturgeon (*Acipenser baerii*), substituting FM protein with 30% RM in their diets did not cause detrimental effects on growth performance, body composition, or blood parameters [41]. Post-slaughter processing of livestock and poultry yields diverse organic waste and byproducts abundant in valuable resources like proteins, fats, keratins, collagen, gelatin, and minerals [42]. Maximizing the utilization of these byproducts from livestock and poultry not only addresses the scarcity of FM resources in aquaculture but also substantially cuts production costs. A study on large sturgeon (*Huso huso*) [43] revealed that PBM, ranging from 23.59% to 25.18%, could effectively substitute FM protein in the large sturgeon diet. Similarly, a report on Siberian sturgeon [44] highlighted that using up to 80% PBM as a replacement for FM did not cause detrimental effects on growth or body composition. Besides conventional plant and animal protein sources, single-cell bio-protein sources have gained substantial attention in formulating new aquaculture feeds due to their nutrient density and favorable pricing. Research suggests that incorporating microalgae as a substitute for 10% of dietary FM does not significantly impact the growth performance of Siberian sturgeon [45]. Another study observed enhanced sturgeon growth when FM was replaced by 50% spirulina in the diet [46].

As the scale of commercial sturgeon aquaculture expands each year, its demand for high-quality compound feeds has increased dramatically. However, the decline in global fishery resources has led to a decrease in the annual production of fishmeal protein and a doubling of its price. The cost of compound feeds for aquaculture has also increased gradually; therefore, it is imperative to find alternative sources of fishmeal protein that meet the market demand. Prior to this, a large number of scholars have carried out experiments on replacing fishmeal with plant proteins such as SM, cottonseed meal, and peanut meal and achieved good results. The development and utilization of cheap, high-quality plant and animal protein sources and the increase of the proportion of these protein sources in aquafeeds can, to a certain extent, effectively help the aquaculture industry to get rid of the dilemma of insufficient fishmeal resources and can reduce the cost of aquaculture, thus improving the economic returns of aquaculture. This experiment aims to evaluate RM as an FM alternative in the diets of hybrid sturgeon (*A. schrenckii* ♀ × *A. baerii* ♂) based on the growth performance, body composition, immunological responses, intestinal histology, and inflammatory response.

## 2. Materials and Methods

### 2.1. Feeding Trial

This study utilized 450 juvenile hybrid sturgeon (*A. schrenckii ♀ × A. baerii ♂*) with similar weights as the experimental cohort, sourced from the Fangshan Sturgeon Breeding Station of the Chinese Academy of Fisheries Sciences. After a 2-week acclimation phase where the fish received the basal diet twice a day, the experiment comprised five groups, each assigned a different proportion of RM substituting FM: 0% FM replacement (G1), 25% FM replacement (G2), 50% FM replacement (G3), 75% FM replacement (G4), and 100% FM replacement (G5). At the acclimatization period's conclusion, the initial fish weight (W0) was recorded at 29.21 ± 2.04 g, and 450 fish were randomly distributed among 15 tanks (300-L tank volume) with 30 fish per tank. These tanks were then randomly assigned to the five groups mentioned above, each with three replicates. The feed formulations and amino acid composition used in the feeding trials are detailed in Tables 1 and 2, respectively, and it is evident that we added methionine (Met), lysine (Lys), and soybean protein concentrate to the feeds so as to be able to formulate diets with isonitrogenous and isoenergetic contents according to the formulations. Over the 12-week feeding trial, fish were fed four times daily (6:00, 10:00, 14:00, and 18:00) with approximately 3% of body weight near satiety, and the weight of feed fed (*Wf*) was recorded. To maintain a stable environment, water quality was monitored daily, with parameters such as dissolved oxygen (5.8–6.0 mg/L), temperature (22.5–23.5°C), pH (7.8–8.0), NO_2_^−^-N (<0.02 mg/L), NH_4_^+^-N (<0.2 mg/L), and a photoperiod of 12 h of natural light.

### 2.2. Sample Collection

At the end of the feeding trial, the fish underwent a 24-h fasting period to achieve complete emptying of the digestive tract. Three fish from each tank were anesthetized with MS-222 (tricaine methanesulfonate, Sigma–Aldrich, St. Louis, MO, USA, 100 mg/L), and then obtained the body weight and length for further analysis. Blood was drawn from the tail vein and spun at 5000 *g* and 4°C for 10 min to gather the supernatant for analyzing plasma biochemical parameters. Subsequently, fish were dissected using sterile instruments, and the viscera and liver from each fish were separated and weighed for the calculation of somatic index. Pyloric appendix, stomach and liver (three fish per tank) were collected and stored in liquid nitrogen for subsequent determination of digestive enzymes and antioxidant enzymes. Nine intestines from each group were separated and removed the mesenteric fat cleanly; the spiral valvula intestine were collected for digestive enzymes analysis, and the duodenums were divided into three segments for histological examination, digestive enzymes, and gene expression analysis. Additionally, three fish were randomly selected from each tank to determine the conventional nutrient content of the whole fish.

### 2.3. Nutrient Content of Whole Fish

The experimental diets and fish underwent analysis following AOAC protocols. Oven-drying samples assessed moisture content at 105°C until a consistent weight was achieved. Crude protein (N × 6.25) analysis employed the Kjeldahl method (2300, FOSS, Sweden) for nitrogen measurement, while ash content determination involved incineration at 550°C for 4 h. Crude lipid content was quantified using the Soxhlet method (Extraction System-811, BUCHI, Switzerland).

### 2.4. Total Amino Acid Determination

According to the protocol of GB/T5009124-2003 [47], 40–50 mg of the dried and defatted FM were measured and placed in a 50 mL ampoule. To this, 10 mL of 6 mol/L hydrochloric acid was added, and the ampoule was sealed and subjected to constant temperature drying at 110 ± 1°C for 22 h. After cooling, the ampoule was opened, and 10 mL of 6 mol/L sodium hydroxide solution was added to adjust the pH value. The hydrolysate was mixed with 0.02 mol/L hydrochloric acid to reach a volume of 100 mL. The resulting hydrolysate and a mixed amino acid standard solution were aspirated to 1 mL, filtered into a moving sample bottle, and analyzed using the Hitachi L-8900 amino acid analyzer. Tryptophan (Trp) underwent pretreatment through alkaline hydrolysis according to NY/T 57-1987, where 40 mg of defatted FM was weighed into a 20 mL stoppered glass test tube. To this, 1 mL of 10% potassium hydroxide was added, and the sample underwent hydrolysis at 40°C for 17 h in a constant temperature oven. After cooling, 0.2 mL of 5% p-dimethylaminobenzaldehyde and 0.2 mL of 1% sodium nitrate were added, followed by shaking and cooling in ice water. Subsequently, 5 mL of concentrated hydrochloric acid was added, and the sample was placed in a constant temperature oven for 45 min.

### 2.5. Liver Enzyme Activity Assays

Liver samples were weighed and homogenized with precooled buffer (0.86% saline) at a 1:9 ratio, followed by centrifugation (7000 *g*, 4°C, 15 min) to obtain supernatant for biochemical analysis. Biochemical assays were performed using commercially available kits sourced from Nanjing Jiancheng Bioengineering Institute, Nanjing, China, following the manufacturer's protocols. Superoxide dismutase (SOD; A001-3-2) activity was determined spectrophotochemically employing the ferricytochrome method, with xanthine/xanthine oxidase as the source of superoxide radicals. The reaction mixture included 50 mM potassium phosphate buffer (pH 7.8), 0.1 mM EDTA, 0.1 mM xanthine, 0.013 mM cytochrome *c*, and 0.024 IU/mL xanthine oxidase. One activity unit was defined as the enzyme quantity needed to induce a 50% reduction in the ferricytochrome *c* rate, measured at 550 nm. Lysozyme (LZM; A050-1-1) content was assessed using turbidimetry. About 0.2 mL supernatant was added to 2 mL bacteria solution with sufficient mixing and then removed to the colorimetric dish to determine the transmittance at 530 nm to calculate the LZM activities. Lipid peroxidation, represented by malondialdehyde (MDA; A003-1-2) equivalents, was analyzed utilizing the thiobarbituric acid reaction method [48].

### 2.6. Intestinal Digestive Enzyme Activity Test

Intestinal samples were weighed and combined with pre-cooled buffer (0.86% saline) at a ratio of 1:9. The resulting supernatant obtained after centrifugation (7000 *g*, 4°C, 15 min) was used for biochemical analysis. All assay kits used were procured from the Nanjing Jiancheng Bioengineering Institute, Nanjing, China, unless specified otherwise. These kits included the trypsin activity assay kit (Protease; A080-2-2), amylase activity assay kit (AMS; C016-1-1), and lipase activity assay kit (LPS; A054-1-1).

### 2.7. Histological Examination

The duodenum was fixed with Bouin'sfixative solution for 24 h and subsequently rinsed several times with running water to eliminate the solution. Following dehydration in a graded ethanol solution (70%–100%), they were immersed in xylene. The blocks were then embedded in paraffin wax at 60°C and sectioned to a thickness of 6 μm using a sectioning machine (Leica-RM2235). These sections were stained with hematoxylin-eosin and sealed with neutral resin. Images capturing the intestinal histomorphology were captured using a light microscope (Nikon, DS-Ri2) and analyzed utilizing Motic Images Plus 2.0 software. For each image, 10 villi were randomly selected, and measurements of intestinal muscle thickness, height, and width were conducted.

### 2.8. Real-Time Polymerase Chain Reaction (PCR) Analysis

The total RNA of midgut extraction was performed using the Total RNA Separation System Kit (TaKaRa, Dalian, China), following the manufacturer's recommended protocol. The concentration of RNA was quantitatively assessed using a spectrophotometer (NanoDrop 2000, Thermo Fisher, Germany). RNA integrity was examined through agarose gel electrophoresis, and the RNA quality was verified by ensuring that the absorbance ratio of A260/A280 nm fell within the range of 1.8–2.0. Subsequently, the PrimeScript RT kit (TaKaRa, Dalian, China) was employed to reverse transcribe RNA into cDNA, following the manufacturer's instructions. Quantitative PCR (qPCR) was conducted in LightCycle 480 Thermal Cycles (Roche, Germany) using LightCycles 480 SYBR Green I Master (Roche, Germany), with a total reaction volume of 10 mL. All amplification reactions were performed in triplicate. The mRNA expression levels of the target gene were corrected based on *β*-actin, and the fold changes were normalized to the level of the control group according to the 2^−ΔΔCt^ method [49]. Prior to qPCR amplification, the efficiency of all the primers was evaluated by previously reported methods to ensure reliability. The experimental conditions are as follows: pre-denaturation at 95°C for 30 s, followed by 40 amplification cycles: denaturation at 95°C for 5 s, annealing at 60°C for 34 s. After the completion of the final amplification cycle, obtain a melt curve by performing steps at 95°C for 15 s, 60°C for 1 min, and 95°C for 15 s to verify the specificity of the amplification. cDNA was used as template DNA for RT-PCR amplification reactions. Three replicates of each amplification reaction were used for comparison. The primers used for the *β*-actin gene and target gene are detailed in Table 3.

### 2.9. Calculations and Statistical Analysis

The calculation formula for the growth performance index was as follows:

  $$\begin{array}{c} \text{Weight gain rate }\left(\text{WGR};\%\right)=100\times \left(W_{t}-W_{0}\right)/W_{0}, \end{array}$$

  
$$\begin{array}{c} \text{Condition factor }\left(\text{CF};\%\right)=100\times W_{t}/L_{t}^{3}, \end{array}$$

  
$$\begin{array}{c} \text{Feed conversion ratio }\left(\text{FCR};\%\right)=W_{f}/\left(W_{t}-W00\right), \end{array}$$

  
$$\begin{array}{c} \text{Specific growth rate }\left(\text{SGR};\%/day\right)=100\times \left(\ln W_{t}-\ln W_{0}\right)/t, \end{array}$$

  
$$\begin{array}{c} \text{Protein efficiency ratio (PER)}=\left(W_{t}-W_{0}\right)/\left(W_{f}\times \text{feed protein content}\right), \end{array}$$

  
$$\begin{array}{c} \text{Survival rate }\left(\text{SR};\%\right)=100\times N_{t}/N_{0}, \end{array}$$

  
$$\begin{array}{c} \text{Hepatosomatic index }\left(\text{HSI};\%\right)=100\times \left(\text{liver weight (g)}/\text{body weight (g)}\right), \end{array}$$

  
$$\begin{array}{c} \text{Viscerosomatic index }\left(\text{VSI};\%\right)=100\times \left(\text{viscera weight (g)}/\text{body weight (g)}\right), \end{array}$$

where *W*_0_ is the initial body weight of the fish (g), *W*_*t*_ is the final body weight (FBW) of the fish (g), *L*_*t*_ is the final body length (cm), *W*_*f*_ is the feed intake (g), *N*_0_ is the initial number (*f*), *N*_*t*_ is the final number (*f*), and *t* is the test days (*D*).

Data were processed and statistically analyzed as follows:

The statistical software SPSS 22.0 for Windows (SPSS Inc., Chicago, IL, USA) facilitated a one-way analysis of variance alongside Tukey's multiple data comparisons. All data were checked for homogeneity of variance using Levene's equality of variances test. Data are presented as mean ± standard deviation, and significance within the group was denoted by *p* < 0.05.

## 3. Results

### 3.1. Growth and Feed Utilization

The impact of replacing various FM portions with RM on sturgeon growth performance is summarized in Table 4. As the RM substitution increased, FBW, weight gain rate (WGR), and survival rate (SR) demonstrated a declining pattern. FBW significantly decreased when RM exceeded 75% substitution (*p* < 0.05), and feed conversion ratio (FCR) notably increased compared to the control group (*p* < 0.05). Additionally, WGR and SGR significantly dropped when RM surpassed 50% substitution (*p* < 0.05). Hence, the study inferred that substituting FM with RM below 50% had a limited impact on growth performance.

### 3.2. Body Indices

The effects of substituting RM for various FM proportions on sturgeon body parameters are detailed in Table 5. There was no notable effect of RM substitution on condition factor (CF) and viscerosomatic index (VSI) (*p* > 0.05). However, the hepatosomatic index (HSI) showed a tendency to decrease with higher RM substitution (*p* < 0.05).

### 3.3. Proximate Composition of Fish

The impact of substituting various FM proportions with RM on sturgeon whole fish composition is presented in Table 6. RM substitution did not significantly affect the moisture and crude lipid content of sturgeon body composition (*p* > 0.05). However, the crude protein and ash content notably decreased with higher levels of RM substitution.

### 3.4. Whole-Body Amino Acids Composition

The effect of RM substitution at various proportions for FM on sturgeon whole fish amino acids is delineated in Table 7. As the proportion of RM replacing FM increased, the leucine (Leu) and Lys content in sturgeon whole fish increased (*p* < 0.05) when the RM substitution exceeded 25% (G2). Furthermore, at a 100% substitution ratio, the levels of Leu and Lys were significantly higher than in the other experimental groups (*p* < 0.05).

Increasing the RM substitution (>50%) for FM notably enhanced the valine (Val) and isoleucine (Ile) contents in the entire sturgeon (*p* < 0.05). These contents significantly surpassed those in other experimental groups at a 100% substitution rate (*p* < 0.05). Moreover, as the RM replaced FM in larger proportions, the threonine (Thr) and Met content in the entire sturgeon across all experimental groups displayed an upward trend (*p* > 0.05). Nonetheless, the levels of Thr and Met surpassed those of the G1 (control group) significantly only at a substitution ratio of 100% (*p* < 0.05). Conversely, the levels of histidine (His) and arginine (Arg) in each experimental group did not display significant differences compared to the control group (*p* < 0.05).

The whole-fish glutamate (Glu), alanine (Ala), tyrosine (Tyr), and proline (Pro) contents in each experimental group of sturgeon exhibited an increase as the proportion of FM replaced by RM rose (*p* < 0.05). The whole-fish Glu content in sturgeon reached its peak at a 50% substitution proportion, significantly surpassing that of control G1 (*p* < 0.05). Furthermore, when the substitution proportion reached 100%, the whole-fish Ala, Tyr, and Pro contents in the sturgeon reached their maximum and were significantly higher than those of control G1 (*p* < 0.05).

The whole fish aspartate (Asp) content in each experimental group exhibited an initial increasing trend, followed by a subsequent decrease as the proportion of FM replaced by RM increased. Specifically, the Asp content reached its highest value at a 25% substitution proportion, significantly exceeding that of the control group (*p* < 0.05). In contrast, the serine (Ser) content in the whole sturgeon reached its lowest value when the proportion of FM substituted with RM increased to 25%, significantly lower than in the other experimental groups (*p* < 0.05). The levels of phenylalanine (Phe) and cysteine (Cys) in the entire sturgeon among all experimental groups remained relatively stable as the replacement ratio of FM with RM increased, except when the substitution ratio hit 100%, resulting in higher Phe and Cys levels compared to other experimental groups (*p* < 0.05).

### 3.5. Liver Antioxidant Activities

The impact of substituting various ratios of FM with RM on the antioxidant indices of sturgeon is presented in Table 8. Notably, at a 50% replacement level of FM with RM, there was an enhancement in the sturgeon liver SOD activities and MDA contents. The MDA activity was significantly higher than that of the control G1 (*p* < 0.05).

### 3.6. Blood Biochemical Parameters

The impact of varying proportions of RM replacing FM on sturgeon's blood biochemical indicators is detailed in Table 9, and with an increase in RM substitution, glutamic acid (GLU), total protein (TP), and albumin (ALB) levels in the blood exhibited an upward trend (*p* < 0.05). Upon reaching a 100% substitution ratio, the blood's alanine aminotransferase (ALT) and aspartate aminotransferase (AST) levels were notably higher than in other experimental groups (*p* < 0.05). Moreover, the ALB content in each experimental group significantly surpassed that of the control group (*p* < 0.05). The triglyceride (TG) content in each experimental group was notably lower than that in the control group G1 (*p* < 0.05). However, no significant differences were noted in cholesterol content among the groups (*p* < 0.05).

### 3.7. Lysozymeactivities in the Intestine

The impact of RM substitution across different FM proportions on the sturgeon's nonspecific intestinal immunity is outlined in Table 10. With an increasing substitution of RM for feed FM, the LZM activity in the pyloric cecum, duodenum, and valve intestine showed a decreasing trend (*p* > 0.05). Upon reaching a 100% substitution ratio, the LZM activity in these areas significantly dropped compared to the control group (*p* < 0.05). Conversely, there were no notable changes observed in LZM activity within the sturgeon's stomach (*p* > 0.05).

### 3.8. Intestinal Digestive Enzymes

The impact of substituting different proportions of FM with RM on the sturgeon's intestinal digestive enzymes is presented in Table 11. As the RM substitution increased, the overall LPS and AMS activities in the duodenum showed a declining trend (*p* > 0.05). However, when the substitution ratio reached 100%, both LPS and AMS activities in the duodenum were significantly lower compared to the control G1 (*p* < 0.05). Similar trends were observed in the sturgeon's flap intestine, with LPS and AMS viabilities decreasing as RM replaced FM. At 100% substitution, both LPS and AMS activities in the flap intestine were notably lower than the control group G1 (*p* < 0.05). Additionally, the maximum sturgeon flap intestine protease vitality was achieved at a 50% substitution ratio.

### 3.9. Intestinal Histology

**Figure 1:**
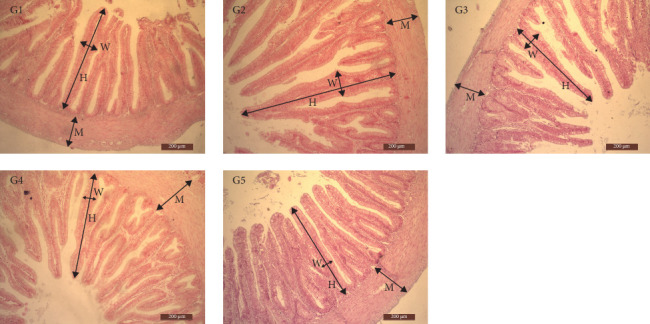
Effects of growth and immune factor gene expression levels. *Note:* The light micrograph of the duodenum of sturgeon fed control (G1) and rapeseed meal replacement (25%, 50%, 75%, and 100% corresponding to G2, G3, G4, and G5, respectively) diets. H, W, and M represent villi height, villi width, and muscle thickness. Scale bar = 200 μm.

The effects of substituting different proportions of FM with RM on the histomorphological structure of the sturgeon duodenum are summarized in Table 12. As the proportion of RM substitution for FM increased, the muscular tunic (MT) of the sturgeon duodenum exhibited a significant decreasing trend (*p* < 0.05), with the basal thickness (MT) reaching its minimum value at 100% substitution ratio. When the proportion of RM substitution exceeded 50%, the villi width (VW) of the sturgeon duodenum showed a significant decreasing trend (Figure 1, *p* < 0.05). Conversely, when the substitution ratio reached 25%, the villus height (VH) of the sturgeon duodenum was significantly higher than that of the control group (*p* < 0.05). However, at a 100% substitution ratio, the VH of the sturgeon duodenum was significantly lower than that of the control group.

### 3.10. Effects of Growth and Immune Factor Gene Expression Levels

**Figure 2:**
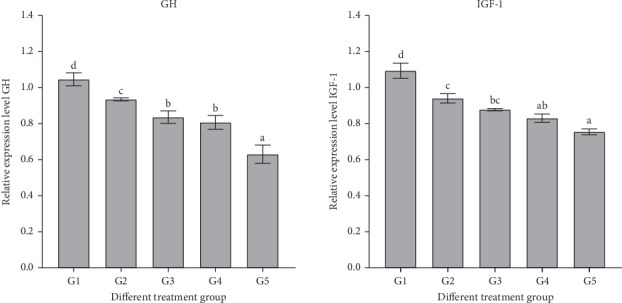
Expression levels of GH, IGF-1 genes in the intestine of sturgeon-fed rapeseed meal. Values are means ± S.E. (*n* = 3). Bars with different letters indicate significant differences (*p* < 0.05; Tukey's multiple range test).

As the ratio of RM replacing FM increased, the intestine showed a significant decrease in the expression levels of GH, IGF-1, Toll-like receptor 1 (TLR1), and IL-10 genes (*p* < 0.05). GH and IGF-1 genes reached their lowest values at 100% replacement, significantly lower than those observed in the control group (Figure 2).

**Figure 3:**
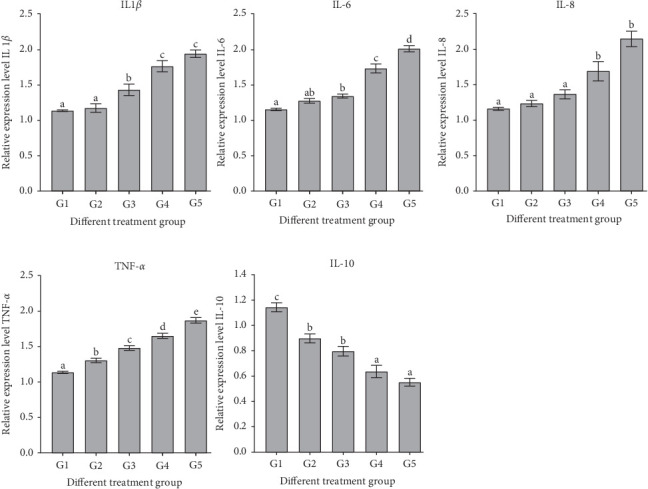
Expression levels of IL1*β*, IL-6, IL-8, TNF-*α*, and IL-10 genes in the intestine of sturgeon fed rapeseed meal. Values are means ± S.E. (*n* = 3). Bars with different letters indicate significant differences (*p* < 0.05; Tukey's multiple range test).

The gene expression levels of IL-1*β*, IL-6, IL-8, and Toll-like receptor 2 (TLR2) in the intestine exhibited a significant increase (*p* < 0.05) with the rising proportion of RM substituting FM, reaching their highest values for IL-1*β* genes at a 100% substitution level, significantly surpassing those of the control group (*p* < 0.05) (Figure 3).

**Figure 4:**
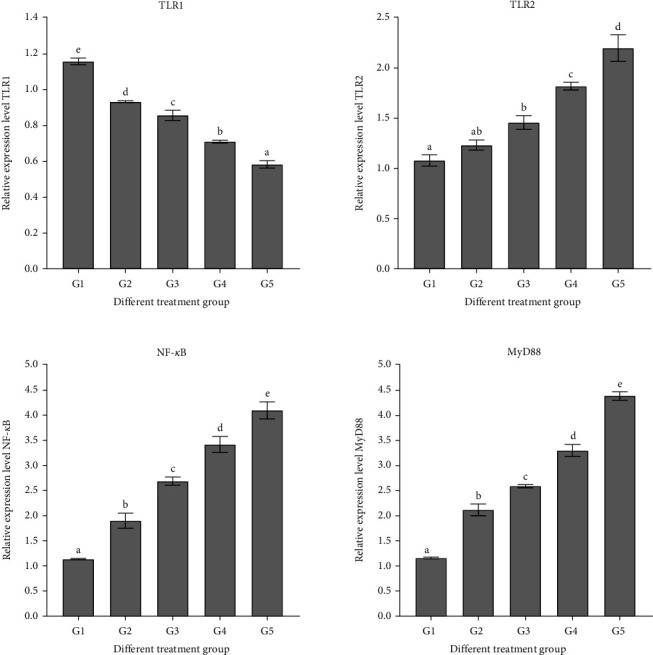
Expression levels of TLR1, TLR2, NF-*κ*B, and MyD88 genes in the intestine of sturgeon fed rapeseed meal. Values are means ± S.E. (*n* = 3). Bars with different letters indicate significant differences (*p* < 0.05; Tukey's multiple range test). MyD88, myeloid differentiation factor 88; TLR1, Toll-like receptor 1; TLR2, Toll-like receptor 2.

As the FM was increasingly replaced by RM, the expression levels of TNF-*α*, NF-*κ*B, and myeloid differentiation factor 88 (MyD88) genes in the intestine significantly increased (*p* < 0.05), with the IL-6 gene expression reaching its peak at a 100% replacement level, significantly surpassing that of the control group (*p* < 0.05) (Figure 4).

## 4. Discussion

Research conducted on cobia (*Rachycentron canadum*) [50] and grass carp [25] observed a decline in growth performance with elevated RM levels in the diet. In this investigation, a negative correlation between growth performance and the proportion of RM in the sturgeon diet was observed. Specifically, the FBW and WGR significantly decreased when RM replaced more than 50% of FM, and the SGR significantly reduced when RM exceeded 100% of FM. These findings align with prior research indicating that incorporating RM into the diet can diminish growth performance, primarily due to the presence of ANFs in RM, which may adversely affect feed palatability, fish growth, and feed utilization, potentially leading to metabolic disruptions and reduced overall fish intake and digestive efficiency [20]. Moreover, as the proportion of RM substitution escalated in the experimental diet, a declining trend in Lys content in the feed was observed (Table 2). This decrease in Lys content may potentially influence the growth performance of sturgeons. In contrast to findings in red snapper [51], where a significant reduction in growth performance was observed only at levels exceeding 75% RM in the diet with yeast fermentation, our study yielded differing results [52]. The reason may be related to whether RM has been subjected to fermentation treatment.

CF, representing the relationship between body weight and body length, serves as an indicator of the animal's body size and growth, with a higher CF suggesting robust body size and rapid weight gain. VSI, denoting the weight of internal organs as a percentage of body weight, reflects organ growth. HSI, representing the weight of the liver as a percentage of body weight, indicates efficient utilization of fat content in the feed. In this study, the addition of RM to the feed did not significantly impact CF and VSI. However, an increase in RM content correlated with a decrease in HSI, potentially attributed to factors such as the hindrance of liver cell development by anti-nutritional components in the feed. In a study involving rainbow trout (*Oncorhynchus mykiss*) [53], the replacement of some FM with SM and rapeseed protein concentrate showed no significant impact on the crude protein and ash content in whole fish and muscle. In contrast, the present study revealed a decreasing trend in crude protein and ash with increasing levels of RM in the diet, deviating from previous findings. However, in a study involving spotted snapper juveniles (*Lutjanus guttatus*) [21], crude protein levels exhibited a linear decrease as the proportion of RM substitution increased. This inconsistency may be attributed to the high fiber content and ANFs in RM, leading to reduced protein digestibility. Additionally, variations in the growth and metabolic capacities of different fish species could contribute to these discrepancies.

At the same time, a consistent association has been established between nutrient digestion, absorption, utilization, and the activity of digestive enzymes in the gastrointestinal tract [54]. Studies on juvenile Asian red-tailed catfish [17, 37] and *P. ussuriensis* [27] have demonstrated a gradual decline in lipase and amylase activities in the gastrointestinal tract, with increasing levels of RM replacing FM. In alignment with these findings, the present study observed a significant reduction in lipase and amylase activities in the duodenum of sturgeon as the percentage of RM substitution increased. Notably, when the substitution rate exceeded 100%, both lipase and amylase activities in the duodenum and spiral valvula of sturgeon were markedly lower than those in the control group and several experimental groups. This pattern was consistent with the observed growth performance, aligning with previous findings. Nevertheless, in a study involving Japanese sea bass (*Lateolabrax japonicus*) [55], no significant impact was noted on lipase and amylase activities in the bass intestine when utilizing double-low RM as a substitute for the FM portion of the diet. This discrepancy may be attributed to the higher concentrations of thioglucoside and erucic acid in RM compared to double-low RM [56], leading to reduced palatability of the test feed. Consequently, this diminished the fish's ability to digest and absorb nutrients from the diet, subsequently affecting the growth of the experimental fish.

Examining the histology of the intestinal tract provides valuable insights into the impact of feed ingredients on fish growth and development [57]. Nutrient absorption primarily occurs through intestinal villi, with their length, width, and thickness serving as crucial indicators of intestinal nutrient absorption [58, 59]. In this experiment, the increase in RM substitution for FM led to a significant decrease in the width of the villi and the thickness of the muscular layer in the sturgeon's duodenum compared to the control group. Certain studies have indicated that the consumption of rapeseed protein feeds in fish may induce intestinal inflammation [60, 61]. The adverse effects of plant protein on the gastrointestinal tract are likely linked to the presence of ANFs [62], and the protein origin in the diet can influence the gut's integrity in fish [63].

SOD serves as a crucial defense mechanism against free radicals [64, 65], while MDA acts as the end product of lipid peroxidation, functioning both biotoxically and as a marker for assessing oxidative stress levels in an organism [66]. In this investigation, there was no significant alteration in SOD content in the sturgeon liver as the proportion of RM substitution increased. Upon reaching a 100% substitution ratio, the SOD content reached its minimum, aligning with findings from a study involving Nile tilapia (*O. niloticus*) and Galileo tilapia (*Sarotherodon galilaeus*) [67]. Upon reaching a 50% substitution rate, the liver's MDA content exhibited a significant increase compared to other experimental groups. In contrast, a study involving Nile tilapia (*O. niloticus*) suggested that substituting FM with processed double-low RM in the diet did not affect the antioxidant status of the fish liver, indicating a divergence from our findings. This inconsistency could be attributed to the negative impact of ANFs in RM on immune and antioxidant functions [17] or variations in experimental species, age, diet composition, and feeding habits.

ALT and AST are pivotal aminotransferases in the liver, crucial for indicating fish tissue damage and amino acid metabolism [68]. At the same time, their activity not only reflects the strength of amino acid metabolism but also indicates the normalcy of liver function. In a study involving Nile tilapia (*O. niloticus*) and Galileo tilapia (*S. galilaeus*) [67], an increase in serum AST and ALT activities was observed with escalating levels of RM in the diet. Consistent with prior research, our study identified a corresponding elevation in AST and ALT levels with an increased proportion of RM in the diet. This may mean that an increase in the content of RM in the feed may increase the risk of liver damage.

Adequate amino acids are essential for animal growth, and protein deficiency may hinder growth later in life [69]. In our study, there was no significant difference in the total amino acid content of the tested fish. Nevertheless, as the proportion of RM substitutes increased, the content of Thr, Val, Lys, His, and Met also increased. Similar findings were observed in pearl gentian grouper [70] and African catfish [69]. Conversely, varied conclusions were drawn in blunt snout bream (*Megalobrama amblycephala*) [68], tropical sciaenid (*Protonibea diacanthus*) [9], and swimming crab (*Portunus trituberculatus*) [71], potentially attributed to differences in amino acid composition among various plant protein sources. Notably, Met emerged as the first limiting amino acid influencing growth performance and feed utilization in fish [72, 73]. Research on Jian carp (*Cyprinus carpio* var. Jian) [74] and juvenile hybrid grouper (*Epinephelus fuscoguttatus* ♀ × *Epinephelus lanceolatus* ♂) [75] revealed that supplementing puffed feed with Met enhances the dietary essential amino acid (EAA) balance, promoting fish growth. In alignment with these findings, our study incorporated Met-supplemented feed, increasing whole fish Met content proportional to the substitution ratio, which played a vital role in sturgeon growth. Previous studies have suggested that GLU contributes to fish freshness, while Ala enhances sweetness [76, 77]. In our investigation, Glu and Ala levels in the whole sturgeon significantly increased with an escalating proportion of RM substitution, akin to observations in largemouth bass (*Micropterus salmoides*) [78], suggesting a potential enhancement of flavor in sturgeon when replacing dietary FM with RM.

Nonspecific immunity plays a pivotal role in the immune defense mechanism of fish when confronted with external stimuli and pathogenic invasions [8, 67]. LZM, a key indicator of nonspecific immune status, serves as a crucial defense factor in fish by disrupting cell walls and inhibiting biofilm production [79]. Previous studies have documented immune system impairment in fish-fed soy protein-rich diets, exemplified in species like Asian red-tailed catfish [65]. In our study, an observed decline in LZM activity in the pyloric cecum, duodenum, and spiral valvular intestine of sturgeon with increasing dietary rapeseed oil content aligns with findings from prior research.

In this study, the gene expression of intestinal GH and IGF-1 in sturgeon exhibited a significant decrease with an increasing proportion of RM substitution, reaching its nadir at a 65% replacement ratio. This pattern parallels findings in Asian red-tailed catfish [6], where RM replaced dietary FM. Nevertheless, results from experiments with Yellow River carp (*C. carpio*) [80], where a mix of rapeseed proteins (RM, cottonseed meal, and peanut meal) substituted for dietary SM, diverged from our observations. GH, acting through GH receptors (GHRs), stimulates the synthesis and secretion of IGF-I, initiating various growth-promoting effects [41, 81]. The growth rates of fish are regulated by the GH and IGF axes [82, 83], and the protein source in experimental diets may influence the expression of GH- and IGF-1-encoded genes [84]. Additionally, factors such as the experimental feeding regimen, diet composition, temperature, and photoperiod have been demonstrated to impact GH and IGF-1 expression [85].‬‬‬‬‬‬

In fish, inflammation constitutes a crucial component of the intestinal immune response, primarily orchestrated by cytokines [86, 87]. TLRs, integral to the nonspecific immune system, play a pivotal role in immune responses by transducing signals of foreign substance invasion to cells [88]. Pro-inflammatory cytokines such as IL-1*β*, IL-6, IL-8, and TNF-*α* are vital contributors to the inflammatory process [89–92], while IL-10 serves as an anti-inflammatory cytokine [93]. In this investigation, a conspicuous rise in gene expression was observed for IL-1*β*, IL-6, IL-8, and TNF-*α* with an escalating substitution ratio, while there was a significant decrease in IL-10 gene expression. This trend aligns with findings in juvenile gentian grouper (*Epinephalus fuscoguttatus* ♀ × *willow grouper* ♂) [94], Japanese seabream (*L. japonicus*) [95], turbot (*Scophthalmus maximus*) [8] snakehead (*Channa argus*) [96], and sandy tip (*Sillago sihama*) [97]. The observed increase in pro-inflammatory cytokines and decrease in anti-inflammatory cytokine levels suggest that RM may induce intestinal inflammation [98]. However, the adverse impact observed in the sturgeon intestine may also be attributed to the presence of ANFs in the substituted dietary feed. Existing literature suggests that TLR1 and TLR2 could play a significant role in the immune defense against bacterial infections [99]. TLR1 and TLR2 are known to be constitutively expressed in various organs and tissues of fish [100, 101]. Interestingly, their intestinal expressions in this study exhibited an opposing trend, akin to findings in Orange spotted-grouper (*Epinephelus coioides*) [63]. The immune response [102] and MyD88 are integral components of the TLR and interleukin 1 receptor-mediated signaling pathway, playing a crucial role in the defensive immune response [103]. The TLR-MyD88-NF-*κ*B signaling pathway has been previously implicated in the induction of enteritis in pearl gentian grouper [94]. In the current study, the gene expression levels of pro-inflammatory factors IL-1*β*, IL-6, IL-8, and TNF-*α* exhibited an upward trend with increasing substitution ratios. Conversely, as the substitution ratios increased, the gene expression of the anti-inflammatory cytokine IL-10 decreased, resulting in significant elevations in the gene expressions of NF-*κ*B and MyD88. This aligns with findings in juvenile blunt snout bream (*M. amblycephala*) [104], gilthead seabream (*Sparus aurata*) [7], and Atlantic salmon (*Salmo salar*). Considering the collective evidence from gut digestive enzymes, intestinal histology, and gene expression levels related to intestinal inflammation, it becomes evident that elevated levels of RM substituting for FM may have adverse effects on sturgeon intestinal health.

## 5. Conclusion

In conclusion, substituting less than 50% of FM with RM in the hybrid sturgeon diet demonstrated beneficial effects on growth performance, whole fish composition, amino acid profiles, liver antioxidant indices, blood biochemical parameters, and intestinal gene expression levels. However, replacing 75% to 100% of FM with RM in the diet negatively impacted intestinal digestive immunity and compromised liver antioxidant function, ultimately resulting in impaired growth performance in the fish.

## Figures and Tables

**Table 1:** Ingredients and approximate composition of the test diets (air-dry basis, %).

Ingredients (%)	Groups				
	G1	G2	G3	G4	G5
Soybean protein concentrate	9	12.5	15	18.5	22
Fish meal^a^	30	22.5	15	7.5	0
Rapeseed meal	0	7.5	15	22.5	30
Wheat middlings	33	29.5	27	23.5	20
Blood meal	6	6	6	6	6
Fish oil^a^	5	5.15	5.3	5.45	5.6
Soybean oil^a^	5	5	5	5	5
Soybean phospholipid	1	1	1	1	1
Calcium dihydrogen phosphate	2	2	2	2	2
Corn protein	6	6	6	6	6
Premix^b,c^	1	1	1	1	1
Methionine	0	0.06	0.11	0.17	0.22
Lysine	0	0.21	0.41	0.61	0.82
Zeolite	2	1.58	1.18	0.77	0.36
Total	100	100	100	100	100
Proximate analysis of basal diet					
Moisture	9.79	9.83	9.73	9.86	9.92
Crude protein	40.51	40.32	40.22	40.01	40.06
Crude lipid	12.03	11.91	11.78	11.92	11.72

^a^Dalong Feed Company, Harbin, China.

^b^The mineral premix contributed the following content per kg of diets: MgSO_4_ · 7H_2_O 2000 mg, KCl 1500 mg, FeSO_4_ · 7H_2_O 1000 mg, CuSO_4_ · 5H_2_O 20 mg, MnSO_4_ · 4H_2_O 100 mg, ZnSO_4_ · 7H_2_O 150 mg, KI 3 mg, NaCl 500 mg, CoCl_2_ 5 mg, Na_2_SeO_3_ 3 mg.

^c^The vitamin premix contributed the following content per kg of diets: VC 100 mg, VE 60 mg, VK_3_ 5 mg, VA 15,000 IU, VD_3_ 3000 IU, VB_1_ 15 mg, VB_2_ 30 mg, VB_6_ 15 mg, VB_12_ 0.5 mg, nicotinic acid 175 mg, folic acid 5 mg, inositol 1000 mg, biotin 2.5 mg, calcium pantothenate 50 mg.

**Table 2:** Amino acid composition of diets (% dry matter).

Indices (%)	Groups				
	G1	G2	G3	G4	G5
Arg	2.13	2.0	1.96	1.90	1.83
His	1.34	1.3	1.28	1.26	1.23
Ile	1.46	1.43	1.39	1.36	1.34
Leu	3.80	3.73	3.63	3.56	3.5
Lys	2.73	2.65	2.54	2.47	2.40
Met	1.28	1.32	1.34	1.38	1.42
Thr	1.60	1.56	1.50	1.47	1.44
Trp	0.48	0.47	0.46	0.46	0.45
Phe	2.00	1.89	1.96	1.95	1.95
Val	2.22	1.80	2.12	2.09	2.06

Abbreviations: Arg, arginine; His, histidine; Ile, isoleucine; Leu, leucine; Lys, lysine; Met, methionine; Phe, phenylalanine; Thr, threonine; Trp, tryptophan; Val, valine.

**Table 3:** Real-time PCR primer sequences.

Genes	Accession number	Forward primer sequences (5′–3′)	Reverse primer sequences (5′–3′)	Length (bp)
MyD88	KU238084.1	CACATGCGTCACTGTCAAGG	AGCATCACCAGCGAACTCAT	85
GH	JX003684.1	AACTCCCCGTCAGCATTCTG	TGGTGAAAACACGGCTCAGA	157
IGF-1	XM_034024782.2	GCTGAGCTTGTGGACACTCT	TCTCCAGTCGCCTCAGATCA	154
TLR1	XM_034911210.1	CCAGCAATGCATTTTCTGACCGTGT	AGTGAGTTGGCGCTGACATCCA	157
TLR2	XM_034014252.2	CTTTGCCTTCACAAACGCGA	CACTGCGAACAAAGTGCTCC	118
IL-8	MK140599.1	CATCCATCCCAGGCAGATC	TTGACCCAGCGGGCAGTT	112
IL-6	XM_033993799.2	TATACCAGCGGGAAGGACGA	GCTGCTGTGCGAGAGGATAT	141
NF-*κ*B	XM_034013617.2	GCACAGCCTGGTTGGAAAG	AGACGCCGAAGTTGTAGCC	179
IL-1*β*	MF818014.1	GTGTGTGATGCTGGAGGTGA	GGCTCAGAGTCACTTGCTGT	197
TNF-*α*	XM_034909934.1	AGGAGCGGTCTCTACTTCGT	TGTGCGACAGATATACGGGC	82
IL-10	AY887900.1	CTACGGCAGTGTCGAAGTGT	TTGGGGTTGTGGAGTGCTTT	189
*β*-Actin	AY649619.1	GTTGTTGACAACGGTTCCGG	TCCTTCTGTCCCATGCCAAC	128

Abbreviations: MyD88, myeloid differentiation factor 88; PCR, polymerase chain reaction; TLR1, Toll-like receptor 1; TLR2, Toll-like receptor 2.

**Table 4:** Growth and feed utilization.

Indices (%)	Groups				
	G1	G2	G3	G4	G5
IBW	29.87 ± 0.21	29.07 ± 0.55	29.37 ± 1.01	28.33 ± 2.11	30.13 ± 1.20
FBW	164.69 ± 15.02^b^	149.48 ± 8.17^ab^	145.45 ± 15.95^ab^	133.02 ± 7.66^a^	129.53 ± 2.51^a^
WGR	451.54 ± 30.09^c^	424.72 ± 13.21^bc^	404.43 ± 12.74^ab^	370.27 ± 13.73^ab^	330.47 ± 14.12^a^
SGR	2.03 ± 0.06^c^	1.90 ± 0.09^bc^	1.84 ± 0.04^ab^	1.78 ± 0.05^ab^	1.73 ± 0.04^a^
FCR	1.49 ± 0.14^a^	1.72 ± 0.16^a^	1.50 ± 0.20^a^	2.39 ± 0.24^b^	2.24 ± 0.10^b^
Survival rate	93.33 ± 3.06^b^	92.00 ± 6.00^b^	88.67 ± 6.11^b^	83.33 ± 4.62^ab^	74.00 ± 7.21^a^

*Note:* Values are represented as mean ± S.E. of pooled data from triplicates per treatment (*n* = 3). Means in the same row with different subscripts are significantly different (*p* < 0.05).

Abbreviations: FBW, final body weight; FCR, feed conversion ratio; IBW, initial body weight; SGR, specific growth rate; WGR, weight gain rate.

**Table 5:** Body Indices.

Items	Groups				
	G1	G2	G3	G4	G5
CF (g cm^−3^)	0.27 ± 0.00	0.26 ± 0.01	0.26 ± 0.02	0.26 ± 0.02	0.27 ± 0.01
VSI (%)	11.86 ± 2.75	13.18 ± 0.86	12.67 ± 1.21	15.64 ± 1.22	12.25 ± 0.48
HSI (%)	3.29 ± 0.79^b^	2.39 ± 0.16^a^	1.86 ± 0.17^a^	1.96 ± 0.12^a^	2.07 ± 0.16^a^

*Note:* Values are represented as mean ± S.E. of pooled data from triplicates per treatment (*n* = 3). The superscript small letters in the same column mean a significant difference at *p* < 0.05.

Abbreviations: CF, condition factor; HSI, hepatosomatic index; VSI, viscerosomatic index.

**Table 6:** Proximate composition of fish.

Items	Groups				
	G1	G2	G3	G4	G5
Moisture (%)	72.40 ± 0.68	71.89 ± 0.52	72.64 ± 0.54	73.11 ± 0.13	73.06 ± 0.49
Crude protein (%)	16.83 ± 0.61^a^	16.62 ± 0.72^a^	18.30 ± 0.68^b^	17.81 ± 0.30^ab^	17.84 ± 0.42^ab^
Crude lipid (%)	9.60 ± 0.23	9.20 ± 0.23	9.47 ± 0.35	9.47 ± 0.13	9.47 ± 0.35
Ash (%)	1.24 ± 0.03^b^	1.23 ± 0.10^b^	1.06 ± 0.02^a^	1.10 ± 0.03^ab^	1.09 ± 0.02^ab^

*Note:* Values are represented as mean ± S.E. of pooled data from triplicates per treatment (*n* = 3). The superscript small letters in the same column mean a significant difference at *p*  < 0.05.

**Table 7:** Whole-body amino acids composition.

Indices (%)	Groups				
	G1	G2	G3	G4	G5
EAA					
Thr	3.06 ± 0.01^b^	2.65 ± 0.08^a^	3.20 ± 0.15^bc^	3.00 ± 0.06^b^	3.45 ± 0.03^c^
Val	3.40 ± 0.01^a^	3.58 ± 0.06^ab^	3.64 ± 0.06^b^	3.63 ± 0.10^b^	3.90 ± 0.02^c^
Met	2.04 ± 0.01^a^	2.29 ± 0.11^ab^	2.44 ± 0.12^bc^	2.18 ± 0.02^ab^	2.71 ± 0.11^c^
Ile	3.42 ± 0.01	3.37 ± 0.06	3.45 ± 0.05	3.45 ± 0.08	3.47 ± 0.06
Leu	6.21 ± 0.04	6.19 ± 0.09	6.27 ± 0.08	6.31 ± 0.12	6.38 ± 0.02
Phe	2.94 ± 0.02	3.09 ± 0.08	3.19 ± 0.09	3.15 ± 0.14	3.19 ± 0.07
Lys	6.01 ± 0.04^a^	6.45 ± 0.18^ab^	6.61 ± 0.16^b^	6.58 ± 0.26^b^	7.15 ± 0.03^c^
His	1.77 ± 0.01^ab^	1.80 ± 0.11^ab^	1.72 ± 0.03^a^	1.74 ± 0.10^a^	1.97 ± 0.02^b^
Arg	4.43 ± 0.04	4.54 ± 0.11	4.46 ± 0.16	4.67 ± 0.05	4.51 ± 0.03
NEAA					
Asp	4.05 ± 0.02	4.20 ± 0.08	4.07 ± 0.09	4.16 ± 0.14	4.01 ± 0.07
Ser	3.34 ± 0.04	3.28 ± 0.18	3.23 ± 0.16	3.37 ± 0.26	3.28 ± 0.03
Glu	11.37 ± 0.12^ab^	11.69 ± 0.04^b^	12.35 ± 0.08^c^	12.18 ± 0.10^c^	11.21 ± 0.18^a^
Gly	4.70 ± 0.01	4.51 ± 0.65	5.12 ± 0.13	4.70 ± 0.65	4.45 ± 0.03
Ala	4.15 ± 0.03^a^	4.32 ± 0.05^b^	4.32 ± 0.10^b^	4.45 ± 0.04^b^	4.49 ± 0.03^b^
Cys	1.08 ± 0.00	1.07 ± 0.02	1.07 ± 0.03	1.06 ± 0.01	1.06 ± 0.01
Tyr	2.73 ± 0.00	2.75 ± 0.04	2.72 ± 0.04	2.69 ± 0.01	2.71 ± 0.01
Pro	2.24 ± 0.01	2.31 ± 0.11	2.32 ± 0.03	2.37 ± 0.10	2.35 ± 0.06
Total	67.97 ± 0.36	68.49 ± 0.34	68.92 ± 0.53	68.97 ± 0.33	68.84 ± 0.61

*Note:* Values are presented as mean ± S.E. (*n* = 3). Values in the same column with different superscript letters are significantly different (*p*  < 0.05).

Abbreviations: Ala, alanine; Arg, arginine; Asp, aspartate; Cys, cysteine; EAA, essential amino acid; Glu, glutamate; Gly, glycine; His, histidine; Ile, isoleucine; Leu, leucine; Lys, lysine; Met, methionine; NEAA, nonessential amino acid; Phe, phenylalanine; Pro, proline; Ser, serine; Thr, threonine; Tyr, tyrosine; Val, valine.

**Table 8:** Liver antioxidant activities.

Items	Groups				
	G1	G2	G3	G4	G5
SOD (U/mgprot)	104.43 ± 8.06^ab^	108.86 ± 8.09^ab^	91.76 ± 16.34^a^	132.53 ± 5.19^b^	80.46 ± 13.59^a^
MDA (U/mgprot)	4.38 ± 0.08^a^	4.74 ± 0.26^a^	3.48 ± 0.23^a^	5.16 ± 0.85^b^	3.94 ± 0.89^a^

*Note:* Values are represented as mean ± S.E. of pooled data from triplicate per treatment (*n* = 3). The superscript small letters in the same column mean a significant difference at *p* < 0.05.

Abbreviations: MDA, malondialdehyde; SOD, superoxide dismutase.

**Table 9:** Blood biochemical parameters.

Indices (%)	Groups				
	G1	G2	G3	G4	G5
GLU (U/L)	1.24 ± 0.05^a^	5.82 ± 0.28^c^	4.34 ± 0.12^b^	4.87 ± 0.17^b^	4.83 ± 0.31^b^
TP (g/L)	16.34 ± 0.38^a^	30.15 ± 1.10^bc^	28.07 ± 1.59^b^	30.10 ± 1.37^bc^	33.08 ± 1.60^c^
ALB (g/L)	7.28 ± 0.35^a^	14.50 ± 0.47^b^	13.21 ± 1.00^b^	12.97 ± 0.19^b^	14.15 ± 0.76^b^
ALT (U/L)	8.00 ± 0.37^a^	10.14 ± 0.51^b^	7.80 ± 0.49^a^	8.50 ± 0.72^b^	12.40 ± 0.68^c^
AST (U/L)	294.18 ± 8.38^a^	291.33 ± 13.43^b^	256.14 ± 15.25^a^	298.80 ± 34.57^a^	382.60 ± 12.70^b^
TG (mmol/L)	14.69 ± 0.85^b^	6.06 ± 0.45^a^	5.20 ± 0.48^a^	5.41 ± 0.34^a^	6.41 ± 0.37^a^
CHOL (mmol/L)	5.44 ± 0.06	5.38 ± 0.08	5.36 ± 0.06	5.46 ± 0.10	5.47 ± 0.06

*Note:* Values are represented as mean ± S.E. of pooled data from triplicates per treatment (*n* = 3). Means in the same row with different subscripts are significantly different (*p* < 0.05).

Abbreviations: ALB, albumin; ALT, alanine aminotransferase; CHOL, cholesterol; GLU, glutamic acid; ST, aspartate aminotransferase; TG, triglyceride; TP, total protein.

**Table 10:** Lysozymeactivities in the intestine.

LZM (U/mgprot)	Groups				
	G1	G2	G3	G4	G5
Pyloric appendix	2.14 ± 0.14^b^	1.55 ± 0.05^ab^	1.12 ± 0.08^ab^	1.68 ± 0.60^ab^	0.74 ± 0.13^a^
Duodenum	1.11 ± 0.10^b^	0.98 ± 0.06^ab^	0.84 ± 0.16^ab^	0.78 ± 0.02^ab^	0.61 ± 0.14^a^
Spiral valvula intestine	4.27 ± 0.75^c^	4.87 ± 0.41^c^	2.21 ± 0.09^ab^	3.43 ± 0.45^bc^	1.77 ± 0.25^a^
Stomach	4.00 ± 0.35	3.93 ± 0.44	4.03 ± 0.45	3.21 ± 0.96	3.40 ± 0.55

*Note:* Values are represented as mean ± S.E. of pooled data from triplicates per treatment (*n* = 3). The superscript small letters in the same column mean a significant difference at *p*  < 0.05.

Abbreviation: LZM, lysozyme.

**Table 11:** Intestinal digestive enzymes.

Indices	Groups				
	G1	G2	G3	G4	G5
LPS (U/mgprot)					
Duodenum	86.85 ± 10.22^b^	60.45 ± 3.71^a^	71.52 ± 3.90^ab^	69.82 ± 5.18^ab^	59.60 ± 5.96^a^
Spiral valvula intestine	71.52 ± 0.00^b^	49.81 ± 1.28^ab^	52.36 ± 1.28^b^	61.30 ± 10.63^ab^	39.17 ± 14.47^a^
AMS (U/mgprot)					
Duodenum	405.56 ± 32.11^b^	361.42 ± 34.68^b^	356.79 ± 15.45^b^	345.06 ± 26.32^b^	173.15 ± 27.15^a^
Spiral valvula intestine	476.54 ± 1.63^c^	427.78 ± 48.30^bc^	382.10 ± 21.92^bc^	346.30 ± 48.34^bc^	232.72 ± 9.34^a^
Protease (U/gprot)					
Duodenum	113.20 ± 7.05	144.85 ± 11.28	135.94 ± 13.73	138.21 ± 12.66	144.79 ± 11.51
Spiral valvula intestines	93.68 ± 13.42^a^	188.75 ± 14.07^b^	203.14 ± 15.20^b^	179.35 ± 3.56^b^	161.46 ± 16.23^b^

*Note:* Values are presented as mean ± S.E. of pooled data from triplicates per treatment (*n* = 3). Values in the same column with different superscript letters are significantly different (*p*  < 0.05).

Abbreviations: AMS, amylase; LPS, lipase.

**Table 12:** Intestinal histology.

Items	Groups				
	G1	G2	G3	G4	G5
Muscular thickness (μm)	314.47 ± 1.51^c^	217.63 ± 6.55^b^	187.05 ± 4.01^a^	202.60 ± 3.90^ab^	185.22 ± 8.12^a^
Villus width (μm)	114.08 ± 5.52^c^	106.83 ± 5.57^bc^	92.85 ± 6.18^b^	76.11 ± 5.01^a^	66.50 ± 3.22^a^
Villus height (μm)	699.81 ± 6.32^b^	1021.41 ± 16.72^c^	684.37 ± 23.76^b^	651.94 ± 6.47^ab^	607.82 ± 14.12^a^

*Note:* Values are represented as mean ± S.E. of pooled data from triplicates per treatment (*n* = 3). The superscript small letters in the same column mean a significant difference at *p*  < 0.05.

## Data Availability

The original contributions presented in the study are included in the article, and further inquiries can be directed to the corresponding authors.
